# Why Do People (Not) Engage in Social Distancing? Proximate and Ultimate Analyses of Norm-Following During the COVID-19 Pandemic

**DOI:** 10.3389/fpsyg.2021.648206

**Published:** 2021-06-23

**Authors:** James O. Norton, Kortnee C. Evans, Ayten Yesim Semchenko, Laith Al-Shawaf, David M. G. Lewis

**Affiliations:** ^1^College of Science, Health, Engineering and Education, Murdoch University, Perth, WA, Australia; ^2^Faculty of Science, Charles University, Prague, Czechia; ^3^Department of Psychology, University of Colorado, Colorado Springs, Colorado Springs, CO, United States; ^4^Lyda Hill Institute for Human Resilience, University of Colorado, Colorado Springs, Colorado Springs, CO, United States; ^5^Centre for Healthy Ageing, Health Futures Institute, Murdoch University, Perth, WA, Australia

**Keywords:** perceived norms, pathogen avoidance, behavioral immune system, conformity, proximate-ultimate, pandemic, COVID-19, public health

## Abstract

COVID-19 has had a profound negative effect on many aspects of human life. While pharmacological solutions are being developed and implemented, the onus of mitigating the impact of the virus falls, in part, on individual citizens and their adherence to public health guidelines. However, promoting adherence to these guidelines has proven challenging. There is a pressing need to understand the factors that influence people’s adherence to these guidelines in order to improve public compliance. To this end, the current study investigated whether people’s perceptions of others’ adherence predict their own adherence. We also investigated whether any influence of perceived social norms was mediated by perceptions of the moral wrongness of non-adherence, anticipated shame for non-adherence, or perceptions of disease severity. One hundred fifty-two Australians participated in our study between June 6, 2020 and August 21, 2020. Findings from this preliminary investigation suggest that (1) people match their behavior to perceived social norms, and (2) this is driven, at least in part, by people using others’ behavior as a cue to the severity of disease threat. Such findings provide insight into the proximate and ultimate bases of norm-following behavior, and shed preliminary light on public health-related behavior in the context of a pandemic. Although further research is needed, the results of this study—which suggest that people use others’ behavior as a cue to how serious the pandemic is and as a guide for their own behavior—could have important implications for public health organizations, social movements, and political leaders and the role they play in the fight against epidemics and pandemics.

## Introduction

The World Health Organization (WHO) declared COVID-19 a pandemic on March 11, 2020 (World Health Organization [WHO], 2020a). Since then, the pandemic has had a profound negative impact on many aspects of human life. At the time of writing, the human toll has surpassed three million lives (World Health Organization [WHO], 2020b). This pandemic resulted in unprecedented economic shutdowns, leaving many countries facing fiscal uncertainty (Australian Bureau of Statistics, 2020). Some countries were initially successful in their efforts to limit the spread of the disease, but many have subsequently faced second and even third waves, with uncertainty and concern remaining about further waves (see Xu and Li, 2020).

Given the societal impact of COVID-19 and the role individual citizens play in curtailing infectious diseases, it is essential to understand the psychological processes involved in people’s adherence to pandemic mitigation guidelines (Bavel et al., 2020). Such an understanding could help scaffold to a more in-depth, comprehensive program of research and inform public policy on prevention strategies. Further, insights into these psychological processes could improve COVID-19 messaging in public health initiatives.

### Methods and Challenges in Managing the Spread of a Pandemic

Current methods for controlling the spread of a pandemic involve developing and implementing pharmacological treatments and vaccines, increasing hygiene practices, risk communication (Taylor, 2019), closures of public places, voluntary/mandated quarantines (Lu et al., 2021), contact tracing, rapid testing, and herd immunity through exposure (a controversial approach currently only tested in Sweden; Anderson et al., 2020; Habib, 2020). While there are a number of promising vaccines currently in production, even the most optimistic projections tell us that we will not be able to achieve population immunity on a global scale (60–80% of the population) by the end of 2021 (Wang et al., 2020). While vaccine programs are rolled out, top-down policies will be important for limiting the spread of COVID-19 (see Wilson, 2020; Lu et al., 2021; Luoto and Varella, 2021), but the efficacy of these policies may rest largely on the choices of individual citizens to adhere (or not) to these guidelines. To facilitate this, the WHO put forward a global action plan aimed at limiting the spread of COVID-19 through individual behaviors (World Health Organization [WHO], 2020c). This plan emphasizes the uptake of non-pharmacological interventions (NPIs; e.g., frequent handwashing, social distancing, and self-isolating when unwell) at the individual level (World Health Organization [WHO], 2020c). However, it is not clear that these guidelines were informed by an understanding of the psychological processes that influence individual-level decisions to adhere (or not) to NPIs. Public uptake of NPIs has been highly variable. Some populations have reported adherence rates as high as 90% (see Lennon et al., 2020), whereas others have reported rates of adherence as low as 67%—indicating that 1 out every 3 individuals is not adhering to guidelines (see Block et al., 2020).

In previous pandemics, the uptake of these NPIs has been an uphill battle for the public health sector (Gilles et al., 2011; Mitchell et al., 2011; Steelfisher et al., 2012). In general, people routinely fail to follow handwashing recommendations (Pfattheicher et al., 2018). During the 2009 swine flu pandemic, people who believed that they were in the “low risk” category for infection were less likely to engage in handwashing (Gilles et al., 2011). Another study conducted at the same time found that less than 10% of people with acute respiratory infections stayed home when symptomatic, and as many as 45% of people reported attending social events because they did not believe they were contagious (Mitchell et al., 2011). Even during highly lethal outbreaks, such as the African Ebola virus epidemic, some families sheltered sick relatives at home instead of sending them to quarantine facilities (Shultz et al., 2015). This trend of mixed adherence is reflected in the COVID-19 pandemic. For example, media in the United States, Germany, New Zealand, Belgium, England, and France have all reported an increase in social gatherings following lockdowns (Ölcer et al., 2020). Even the WHO has acknowledged that advising sick people to remain home during a pandemic may be impractical and frequently ineffective (World Health Organization Writing Group, 2012).

These issues highlight the need to gain a better understanding of the predictors of NPI adherence and non-adherence. In the context of a highly transmissible and dangerous virus like that responsible for COVID-19 (Petersen et al., 2020), one individual’s non-adherence to NPIs could have widespread negative effects for others. Public health messaging has focused on this fact as a key motivator in NPI adherence, a sentiment reflected in the prominent WHO campaign message “Protect yourself and others” (World Health Organization [WHO], 2020d). However, the effectiveness of this approach depends on people’s desire to minimize the collective harm of COVID-19. Such a strategy may be somewhat naïve in the face of evidence suggesting that collective harm minimization tends to be a weak motivator for behavior change (Markowitz and Shariff, 2012; Yong and Choy, 2021). Consequently, there is a scientific and public health need for research that identifies predictors of adherence to NPIs during the COVID-19 pandemic. Work has already begun on this front (e.g., see Corpuz et al., 2020; Dinić and Bodroža, 2021; Varella et al., 2021; Yong and Choy, 2021), but a collective effort by researchers working in parallel is needed to rapidly map the individual difference, situational, and other psychological and environmental variables that explain why some people adhere to NPIs, whereas others do not.

One particularly important factor may be perceived social norms (Bavel et al., 2020). This factor may help explain, at a proximate level, why adherence is so mixed: perceived norms can vary substantially across individuals (see Miller and Prentice, 1996). We are unaware of any research, to date, that has investigated perceived norms as a potential explanation for NPI adherence (or non-adherence) during the COVID-19 pandemic. To address this gap in the literature, the present study investigated (1) the relationship between perceived norms and people’s adherence to NPIs and (2) several plausible psychological processes that might be responsible for a link between people’s perceptions of others’ adherence and their own decisions to adhere (or not).

To gain a more in-depth understanding of norm-following behavior, our investigation considers both the proximate and ultimate levels of analysis. Currently, social psychology literature tends to explain norm-following behavior (e.g., conformity) only in proximate terms (e.g., features of the immediate social context) but offers little in the way of ultimate explanation (i.e., why people have cognitive systems for processing and responding to information from the social environment in that way in the first place) (for an in-depth discussion of proximate/ultimate explanations, see Scott-Phillips et al., 2011; Lewis et al., 2017; Al-Shawaf et al., 2019).

### Hypothesis 1: People Mimic Perceived Normative Behavior

It is well-established that social norms influence people’s behavior (Miller and Prentice, 1996), including in the context of disease prevention. Evidence suggests that people’s frequency of engaging in specific disease-prevention behaviors is associated with their perceptions of their peers’ frequency of engaging in those behaviors (Dickie et al., 2018). Research also suggests that social networks can amplify the spread of beneficial as well as harmful health behaviors during epidemics (Christakis and Fowler, 2013). Together, these pieces of evidence suggest that people’s perceptions of others’ adherence may influence their own adherence to NPIs during the COVID-19 pandemic. This is the overarching hypothesis of the current study: people mimic behavior that they perceive as normative (Hypothesis 1). This leads to the prediction that people’s current adherence to NPIs should be positively associated with their perceptions of others’ adherence (Prediction 1.0).

However, if this is correct, *why* does it happen? That is, what are the ultimate origins of such norm-following behavior? Here, we present several alternative, but not necessarily mutually exclusive, mechanisms that could be responsible for a link between people’s perceptions of norms and their own behavior.

### Hypothesis 1.1: The Threat of Social Exclusion Influences People’s Adherence to NPIs

A prominent finding in social psychology is that violation of social norms invites direct or indirect punishment (Perreau De Pinninck et al., 2007; Rudert et al., 2019). One particularly effective form of punishment is ostracism of those who deviate from group norms (Rudert et al., 2019). Social exclusion is a physiologically harmful and psychologically distressing experience for the target (Williams, 2007; Nezlek et al., 2012). In ancestral small-scale hunter-gatherer contexts, ostracism from the group would often have been deadly (Spoor and Williams, 2007; Williams, 2007). Consequently, selection may have favored a “detection system” capable of identifying the threat of ostracism and preventing it from occurring (see Spoor and Williams, 2007; Wesselmann et al., 2012). Consistent with this proposal, people take action to avoid social exclusion whenever possible (Wesselmann et al., 2012).

A consideration of the social group living conditions thought to characterize much of our species’ evolution can also offer insight into why people are ostracized for norm violations in the first place. In ancestral environments, norms of cooperation were essential to group survival (Wesselmann et al., 2012). Many deviations from these norms (e.g., free riding on the benefits of collective efforts) would have been detrimental to other group members (Wesselmann et al., 2012). The proposed ultimate function of ostracism—to punish norm-violators who are inflicting fitness costs on other group members, including oneself—may help explain why neutral observers tend to regard ostracism of norm violators as legitimate when said ostracism serves the benefit of the group (Williams et al., 1998).

In the context of COVID-19, NPI adherence can be conceptualized as a public goods game (see Yong and Choy, 2021). All benefit from others’ adherence, but those individuals who themselves do not adhere to NPIs reap these benefits without incurring the costs of adherence: they are free riders. High levels of adherence to NPIs are crucial to their efficacy; a single individual who engages in such free-riding behavior by not following NPI guidelines can undermine the collective effort. If the psychological mechanisms responsible for ostracizing others marginalize those individuals who violate norms to the detriment of other group members, then those members of society who do not align their behavior with the coordinated actions of others may face the threat of ostracism.

In turn, the threat of being ostracized for disrupting group coordination is a key activator of the shame system (see Robertson et al., 2014). The human shame system operates like a sentinel, vigilantly scanning for cues that indicate the threat of social devaluation (Robertson et al., 2018) and triggering a suite of cognitive, affective, and behavioral responses to mitigate devaluation (see Sznycer, 2019 for a comprehensive description of shame as an internal, behavior-regulating emotion). In the context of the COVID-19 pandemic, if people perceive adherence to NPIs to be the group norm, and the threat of being ostracized for violating group norms activates the shame program, then we should expect people to anticipate experiencing shame for not following NPIs (see Robertson et al., 2014).

If this hypothesis (Hypothesis 1.1) is correct, there should be a positive association between (1) people’s perception of others’ adherence to NPIs and the shame they anticipate they would experience if they failed to adhere (Prediction 1.1.1), as well as between (2) people’s anticipated shame and their reported adherence to NPIs (Prediction 1.1.2). This hypothesis also yields one more prediction: people’s anticipated shame for non-adherence will partially mediate the relationship between their perceptions of others’ adherence and their own adherence (Prediction 1.1.3).

However, the ostracism-avoidance hypothesis is not the only possible explanation for why individuals may match their behavior to what others are doing. A second possibility is that people may use others’ behaviors as an indication of what is “right” or moral.

### Hypothesis 1.2: People Regard Normative Behavior as a Cue to What Is Moral, and This Influences Their Adherence to NPIs

Evolutionary analyses of moral psychology suggest that certain facets of moral cognition serve to coordinate side-taking in disputes, and that these cognitive systems use other people’s public behaviors to help determine which side they will take (see DeScioli and Kurzban, 2013). At an ultimate level of analysis, knowing which side others will take is key. Being on the wrong side—on this view, the minority side—of a dispute could have had considerable negative fitness consequences (e.g., ostracism from the group, punishment, or death; DeScioli and Kurzban, 2013). By contrast, being on the side of the majority—that is, adhering to the group norm—would have helped immensely in avoiding these fitness costs (see Bocian and Wojciszke, 2014).

At an ultimate level of analysis, this suggests that certain facets of human moral cognition may have evolved to take, as input, other people’s positions on an issue in order to regulate one’s own behavior in a manner that safely avoids the costs associated with violating group norms. At a proximate level of analysis, this suggests that these moral cognitive systems will (a) take, as input, others’ behavior and (2) produce, as output, perceptions of morality that track these perceived group norms and motivate behavior to align with these norms.

If this line of reasoning is correct, then (1) there should be an association between people’s perceptions of others’ adherence to NPIs and their perceptions of the moral wrongness of non-adherence (Prediction 1.2.1), (2) there should be an association between people’s perceptions of the moral wrongness of non-adherence and their own adherence (Prediction 1.2.2), and (3) the predicted relationship between people’s self-reported adherence and their perceptions of others’ adherence will be at least partially mediated by perceptions of the moral wrongness of non-adherence (Prediction 1.2.3).

### Hypothesis 1.3: People Use Others’ Behavior to Gauge Disease Severity

Another possibility is that people observe others’ rate of adherence to NPIs and use this information as a cue to the severity of disease threat. Such an explanation would be in line with social learning theory; one of the core principles of Vygotsky’s (1978) work was the idea that others have information that we ourselves do not have, and contemporary social learning theory research highlights the importance of observation-based acquisition of knowledge (Csibra and Gergely, 2007). This perspective from social learning theory—which emphasizes the proximate level of explanation—is compatible with evolutionary reasoning focused on ultimate-level explanations. The parasite stress theory of sociality (Thornhill and Fincher, 2014) posits that humans possess a suite of social tactics to minimize the risk posed by pathogens in the local environment. This theory may shed light on why people would imitate others’ behavior in the context of a pathogen threat. In the framework of this theory, people may use information about the behavior of other members of their group in order to gauge the severity of the disease threat (see Navarrete and Fessler, 2006).

Hypothesis 1.3 is thus that people use others’ behavior as a cue to disease severity, and therefore as a guide for their own behavior. If this is correct, then (1) there should be an association between people’s perception of others’ adherence and their perception of the severity of COVID-19 (Prediction 1.3.1), (2) there should be a relationship between people’s perceptions of the severity of COVID-19 and their own adherence (Prediction 1.3.2), and (3) the relationship between people’s perception of others’ adherence and their own adherence should be, at least, partially mediated by perceptions of the severity of COVID-19 (Prediction 1.3.3).

### The Current Study

**FIGURE 1:**
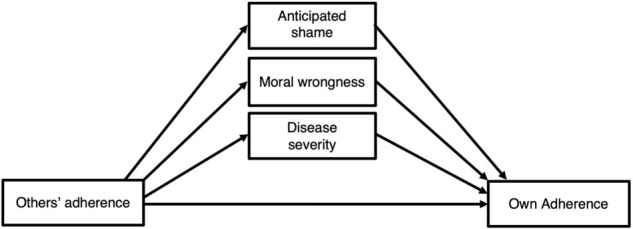
Conceptual model illustrating possible pathways from perceived social norms to NPI adherence.

The current study sought to (1) determine whether people’s perceptions of others’ adherence predict their own adherence, and (2) identify the pathways that might mediate this relationship: through the threat of social exclusion, through perceptions that normative behavior is morally right, or through perceptions of disease severity (Figure 1).

## Materials and Methods

### Ethics Statement

This study was approved by the Murdoch University Human Research Ethics Committee (Approval 2020/049).

### Participants

One hundred seventy-three participants enrolled to participate in the study between June 6, 2020 and August 21, 2020. Participants ranged in age from 18 to 75 (*M* = 33.05, *SD* = 16.22), and represented all but one state and one territory in Australia (59% from Western Australia, 28% from Victoria, 6% from New South Wales, 4% from Queensland, and 1% from the Australian Capital Territory; three participants (2%) did not indicate their state of residence). Participants were recruited through the Murdoch University research participant portal, advertising on social media (Facebook), and snowball sampling. Participants who completed the survey through the Murdoch University research participant portal were granted partial course credit. Participants who were recruited through social media and snowball sampling were not provided any compensation.

### Questionnaire and Procedure

As part of a longer survey investigating the psychological antecedents and consequences of the COVID-19 pandemic, participants completed the measures below via an online questionnaire on the Qualtrics XM platform. The survey was optimized for mobile phones as, given strict lockdowns, computer access may have been limited for participants from low socio-economic backgrounds and those who did not have access to a personal device at home (Raza et al., 2017).

The primary variables measured in the current study were participants’ self-reported adherence to NPIs, perception of others’ adherence to NPIs, perceptions of the seriousness of COVID-19, anticipated shame for non-adherence to NPIs, and perceptions of the moral wrongness of non-adherence. Participants were asked about four NPIs: handwashing, social distancing, quarantining following a positive COVID-19 test, and quarantining following a housemate’s positive COVID-19 test. These four NPIs represent the recommendations put forward in the WHO’s action plan (World Health Organization [WHO], 2020d); the WHO’s revision to include mask wearing occurred after data collection began.

#### Others’ Adherence

To measure perception of others’ adherence, participants were asked to rate what percentage of the people in their country they thought were following NPIs. The exact wording used was *What percentage of people in your country do you think are now following the recommendations below*? For each of the four NPIs, participants answered on a percentage scale of 0–100% (of the population).

#### Own Adherence

To measure current adherence to NPIs, participants were asked to rate how strictly they were currently following the NPIs. The exact wording of this item was: *How strictly are you now following the recommendations below*? Participants provided answers on a 7-point Likert-type scale (1 = Never, 7 = Always). For these questions, participants were only asked about handwashing and social distancing, as only a small fraction of the population of interest (i.e., Australia) was advised to self-quarantine (e.g., when returning from international travel, experiencing flu-like symptoms, awaiting a result for a COVID test, or having had direct contact with someone with COVID-19).

#### Disease Severity

To measure participants’ perceptions of the seriousness of COVID-19, they were asked: *How serious do you think COVID-19 is for public health now*? (1 = Not serious at all, 7 = Very serious).

#### Anticipated Shame

We modeled our measure of anticipated shame on Sznycer et al. (2018)’s cross-cultural study in which they presented participants with a series of scenarios and asked participants to “indicate how much shame you would feel if you were in these situations.” The exact wording we used was: *How much shame do you think you would experience for doing this*? Participants responded on a 7-point Likert-type scale (1 = No shame at all, 7 = A great deal of shame).

#### Moral Wrongness

Following anticipated shame, participants were asked how morally wrong they perceived non-adherence to be. We based our measure of moral wrongness on DeScioli et al. (2011), who asked participants to rate the moral wrongness of specific behaviors on a 7-point scale ranging from 1 (not morally wrong at all) to 7 (very morally wrong). The exact wording we used was: *How morally wrong is this behavior*? (1 = Not morally wrong at all, 7 = Extremely morally wrong).

### Recruitment Time Frame

Data collection for the present study occurred between June 6, 2020 and August 21, 2020.

### Data Preparation and Analysis

Participants’ data were excluded if they did not complete the full questionnaire (*n* = 12) or if they completed the survey in < 450 s (i.e., the time it would take to click through the survey without reading the questions) (*n* = 3). We also only included participants who indicated that they were currently living in Australia. This was because study measures were based on the NPIs being recommended at the time of data collection by the Australian government; these NPIs may not have included NPIs being mandated in others countries (e.g., mask wearing) as well as because of between-country differences in the distance specified for social distancing (e.g., 1.5 m vs. 6 ft). For these and other reasons, the data from six participants who indicated that they were living in a country other than Australia were not included in study analyses. These data preparation procedures yielded a final sample size of 152 participants. Missing data was excluded on a case-by-case basis because the different scales were measuring distinct constructs (Kang, 2013).

For data analysis, we focused exclusively on the NPI of social distancing. This was for several reasons. First, the questionnaire did not collect data on participants’ self-reported adherence to self-quarantine. Second, although handwashing was one of the NPIs, it was a behavior that individuals would have engaged in prior to the pandemic. Without knowledge of participants’ pre-pandemic frequency of handwashing, it would have been difficult to determine to what extent their current handwashing frequency reflected NPI adherence rather than their behavioral and personality patterns unrelated to the pandemic. Conversely, social distancing was a novel behavior specifically prescribed as an NPI for mitigating the impact of the COVID-19 pandemic; unlike handwashing, participants would not have engaged in this behavior prior to the pandemic, so there was no need to control for baseline differences between participants in their pre-pandemic frequencies of social distancing. For these reasons, we focused specifically on the NPI of social distancing.

## Results

All analyses were conducted using IBM Statistical Package for Social Sciences (SPSS) (Version 27). The statistical significance threshold was set at *p* < 0.05. Shapiro-Wilk tests revealed that participants’ self-reported adherence (*p* < 0.001), anticipated shame for non-adherence (*p* < 0.001), perceptions of the moral wrongness of non-adherence (*p* < 0.001), and perceptions of disease severity (*p* < 0.001) all violated the assumption of normality. We therefore used Kendall’s τ for all bivariate correlational analyses.

### Do People Mimic Norms During a Pandemic?

The central hypothesis of the study, Hypothesis 1, was that people match their behavior to perceived social norms. If this is correct, then participants’ current adherence to social distancing guidelines should be positively associated with their perceptions of others’ adherence (Prediction 1.0). In support of this hypothesis, there was a positive association between people’s perceptions of others’ adherence to social distancing recommendations (*M* = 53.74, *SD* = 25.42) and their own adherence (*M* = 5.19, *SD* = 1.85), τ = 0.40, *p* < 0.001, two-tailed, *N* = 149.

### Do People Mimic Norms Due to the Threat of Social Exclusion?

Hypothesis 1.1 was that people follow norms out of concerns about exclusion or ostracism. If this is correct, then there should be positive associations (1) between participants’ perceptions of social norms and their anticipated shame for non-adherence (Prediction 1.1.1), as well as (2) between participants’ anticipated shame and their self-reported adherence (Prediction 1.1.2).

Self-reported adherence was positively linked to anticipated shame (*M* = 4.85, *SD* = 1.83), τ = 0.33, *p* < 0.001, two-tailed, *N* = 147, but there was no relationship between anticipated shame and perceptions of social norms, τ = 0.09, *p* = 0.12, two-tailed, *N* = 147. Collectively, this suggests that people’s adherence may be motivated by a desire to avoid the feeling of shame, but this feeling of shame is independent of violation of perceived social norms.

### Do People Use Normative Behavior to Gauge What Is “Moral”?

Hypothesis 1.2 was that people might use social norms to gauge what is moral. If this is correct, then there should be positive associations (1) between participants’ perceptions of others’ adherence and perceptions of the moral wrongness of non-adherence (Prediction 1.2.1), as well as (2) between participants’ perceptions of the moral wrongness of non-adherence and their self-reported adherence (Prediction 1.2.2).

The findings with respect to morality parallel those observed for shame. There was a positive association between participants’ self-reported adherence and their perceptions of the moral wrongness of non-adherence (*M* = 4.80, *SD* = 1.82), τ = 0.28, *p* < 0.001, two-tailed, *N* = 148, but there was no association between participants’ perceptions of the moral wrongness of non-adherence and their perceptions of others’ adherence, τ = 0.08, *p* = 0.18, two-tailed, *N* = 148. This suggests that people’s adherence may be motivated by perceptions of morality, but these perceptions do not appear to be related to what other people are doing.

### Do People Use Other People’s Behavior as an Indicator of Disease Severity?

Hypothesis 1.3 was that people use others’ behavior as an informative cue to the seriousness of the disease. If this is correct, then there should be associations (1) between participants’ perceptions of others’ adherence and their perception of the severity of COVID-19 (Prediction 1.3.1), as well as (2) between participants’ perception of the severity of COVID-19 and their own adherence (Prediction 1.3.2).

Both results were consistent with this hypothesis. Participants’ perceptions of others’ adherence were positively associated with their perceptions of the seriousness of COVID-19 (*M* = 5.62, *SD* = 1.76), τ = 0.19, *p* = 0.001, two-tailed, *N* = 148, which were positively associated with participants’ own reported adherence levels, τ = 0.36, *p* < 0.001, two-tailed, *N* = 148. This suggests that people’s adherence is partially motivated by their perception of how serious COVID-19 is, a perception that itself is derived partly from observing others’ behavior.

### Mediating Effects of Shame, Moral Wrongness, and Disease Severity

These bivariate analyses lend support to Hypotheses 1 and 1.3. First, people’s adherence to social distancing guidelines track their perceptions of others’ adherence, supporting Hypothesis 1. Second, supporting Hypothesis 1.3, people appear to use others’ behavior as an indicator of the severity of the disease, and therefore as a guide for their own behavior.

**FIGURE 2:**
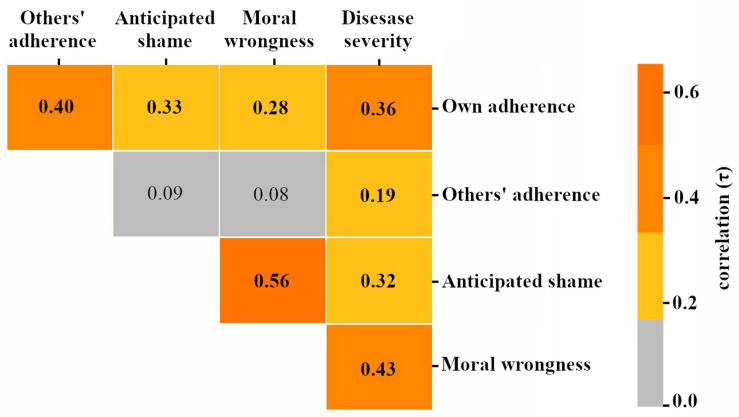
Bivariate correlations between study variables of interest. Note: gray cells indicate non-significant (*p* > 0.05) correlations. All colored cells indicate significant (*p* < 0.05) correlations.

These results suggest that the effect of norms on adherence may be mediated by perceptions of disease severity. However, bivariate correlational analyses cannot directly address mediation. Moreover, we observed multiple inter-correlations between potential mediators (see Figure 2, which displays all bivariate relationships between all study variables of interest).

To more directly examine mediation and produce estimates of the direct and indirect effects of perceived social norms on adherence, we conducted mediation analyses using Hayes (2017) PROCESS macro for SPSS. To control for the observed statistical overlap between the potential mediators (Figure 2) and thereby isolate their independent effects, we included all mediators concurrently (as illustrated in Figure 1)^1^ and used a backward stepwise approach to determine the final model. All analyses employed a 95% CI and 5000 bootstrap as recommended by Hayes (2009). Our final sample size was sufficient to detect a moderate effect following SPSS PROCESS bias-corrected bootstrapping (Fritz and Mackinnon, 2007).

**FIGURE 3:**
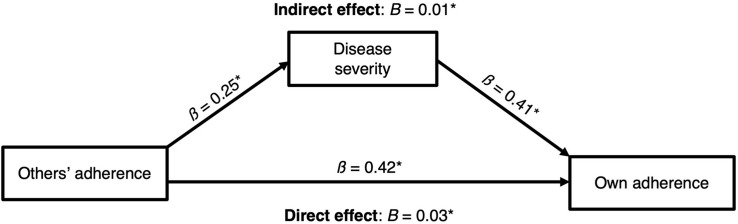
Final mediation model with direct and indirect effects of perceived norms on adherence. *p < 0.05.

This procedure resulted in a final model (Figure 3) that included a significant indirect effect of norms on adherence through perceptions of disease severity, *ab* = 0.007, *SE* = 0.003, 95% BootCI [0.002, 0.014], as well as a direct effect of norms on adherence, *B* = 0.031, *SE* = 0.005, 95% CI [0.021, 0.040] (indirect effect VAF = 0.18).

#### Alternative Mediation Model

In our *a priori* model (Figure 1), we conceptualized the mediators (anticipated shame, perceptions of moral wrongness, and perceptions of disease severity) as *alternative* mediating pathways. However, one potential alternative would be for these psychological variables to have a serial relationship. Here, we describe and test this model. In the serial processing model, people could use others’ adherence as an indicator of the magnitude of disease threat. High levels of adherence among others would cue high levels of disease severity, which would indicate that one could cause great harm to others by not adhering. Because non-adherence could cause such harm to others, moral cognitive systems could produce the perception that non-adherence is immoral or wrong^2^. In turn, the perception that non-adherence is morally wrong—and therefore likely to be met by devaluation from others—could activate the shame program. The shame program would then produce high levels of anticipated shame for non-adherence and thereby motivate adherence.

**FIGURE 4:**
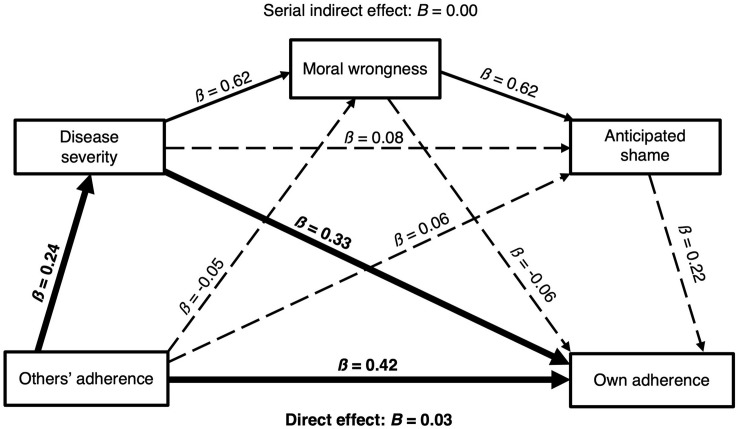
Alternative serial mediation model. Solid lines indicate significant (*p* < 0.05) coefficients. Dashed lines indicate non-significant coefficients. Bolded lines indicate the significant pathways to individuals’ adherence to social distancing guidelines.

We tested this alternative model but did not find support for serial mediation (Figure 4). Specifically, the indirect effect of norms on adherence via the serial pathway through perceptions of disease severity, perceptions of moral wrongness for non-adherence, and anticipated shame for non-adherence was not significant (*a_1_d_2__1_d_3__2_b_3_* = 0.0015, *SE* = 0.0010, 95% BootCI [-0.0001, 0.0040]). In fact, among the seven indirect pathways in this model from people’s perceptions of others’ adherence to their own adherence, only one exhibited a significant effect: the pathway from perceived norms to adherence through perceptions of disease severity—precisely the effect we observed in our *a priori* model (Figure 4).

We recognize that there may be other possible alternative models as well. However, thus far we have tested two conceptually sound models, and have found support for only one mediating pathway, which remained robust across both the *a priori* model and the alternative model: from perceived norms to adherence through perceptions of disease severity.

## Discussion

This study addressed two main questions: first, do people conform their social distancing behavior to what they think others are doing? The answer appears to be yes. Second, why do they do this? We tested several processes by which this might occur and found that people seem to perceive others’ behavior as an informative cue to disease severity, and this in turn influences the extent to which they conform to public health guidelines.

### Threat of Devaluation Does Not Appear to Explain the Relationship Between Norms and Adherence

Our findings indicated a relationship between people’s adherence and their anticipated shame for non-adherence, but no relationship between participants’ anticipated shame and their perceptions of social norms. These findings suggest that the emotion of shame may motivate adherence, but that we do not calibrate shame to our perceptions of others’ adherence. These results tentatively falsify Hypothesis 1.1; the threat of social exclusion or ostracism does not appear to explain the relationship between social norms and people’s adherence.

### Perceptions of Moral Wrongness Do Not Appear to Explain the Relationship Between Norms and Adherence

We observed a link between people’s perceptions of the moral wrongness of non-adherence and their own adherence (Prediction 1.2.2), but there was no relationship between people’s perceptions of social norms and their judgments of the moral wrongness of non-adherence (Prediction 1.2.1). This suggests that perceptions of what is morally right may motivate people to follow social distancing guidelines, but people’s perceptions of the moral wrongness of non-adherence do not appear to be based on what others are doing (or not doing). These results tentatively falsify Hypothesis 1.2.

### Learning About Disease Severity Through the Actions of Others

Study findings were consistent with all three predictions generated from Hypothesis 1.3, providing preliminary evidence that people use others’ behavior as a cue to disease severity, and, in turn, as a guide for their own behavior.

However, perceived disease severity only partially mediated the relationship between participants’ perception of social norms and their adherence. This means that more remains to be discovered about the association between perceived social norms and NPI adherence.

### Explanations and Alternative Interpretations of Study Findings

The current study offers preliminary findings suggesting that perceived social norms may play an important role in people’s psychological and behavioral responses to a pandemic, and therefore need to be better understood in order to mitigate the impact of epidemics and pandemics. Nonetheless, these results should be interpreted tentatively, as the current study had several limitations that should be addressed in future research.

#### Why Is Shame Not Linked to Social Norms?

The finding that shame for non-adherence was unrelated to perceived social norms does not appear to conform to the existing literature (e.g., Sznycer et al., 2016, 2018; Sznycer, 2019). One possibility is that anticipated shame was not an appropriate operationalization of concern about social devaluation. However, our anticipated shame measure was virtually identical to that used in research that showed a high degree of correspondence between shame and social devaluation in both a Western (Sznycer et al., 2016) and a large cross-cultural sample of small-scale societies (Sznycer et al., 2018). One possible explanation for the apparent discrepancy between the current study and previous literature is that the latter used evolutionarily relevant (and non-novel) behaviors, whereas we focused on the behavior of social distancing, which—at least in its very precisely prescribed sense—exhibits evolutionarily novel features. Although ancestral humans likely had behavioral strategies for pathogen avoidance that involved physical distancing, it is less plausible that there would have been precisely specified distances associated with such behavior, which contrasts with formal guidelines during the COVID-19 pandemic (e.g., to maintain a distance of at least 1.5 m from others). It is unlikely that our minds evolved to perceive a meaningful difference between (a) an individual approaching another and stopping at a distance of 1.6 m, and (b) an individual approaching another and stopping at a distance of 1.4 m. These behaviors would have been virtually identical, in function, in ancestral environments, and it is unlikely that selection would have shaped the human mind to perceive them as being substantively different—despite one of them being considered adherence to, and the other a violation of, COVID-19 social distancing guidelines.

#### Why Are Perceptions of Morality Not Linked to Social Norms?

A similar line of reasoning may help explain the absence of a relationship between perceived norms and perceptions of the moral wrongness of non-adherence. Moreover, behaviors that spread COVID-19, such as not socially distancing, appear to lack the properties that activate disease-avoidance systems (Ackerman et al., 2018; Seitz et al., 2020) or trigger automatic emotional reactions, which can be important drivers of moral judgment (Greene, 2001).

COVID-19 is abstract, invisible to the naked eye, and seemingly disconnected from the actions that proliferate it. We do not see or feel the moment COVID-19 transmits from one person to another, and by the time symptoms start showing—if they manifest at all; infected individuals can be contagious without exhibiting any symptoms (see Cheng et al., 2020; Huff and Singh, 2020; Tindale et al., 2020)—the person who spread the virus is often far away and several days have passed since the infection event (Varella et al., 2021). Not only is disease transmission not directly observable, but cues that our mind is likely to process as increasing the risk of transmission, such as physical contact or coughing, are not required for transmission to occur. These properties of the disease and its transmission may make it harder for non-adherent behaviors to trigger moral judgment and condemnation.

Intention also plays an essential role in moral judgment; if a moral transgression is unintentional, it is typically judged much less harshly than intentional transgressions (DeScioli et al., 2011; DeScioli and Kurzban, 2013; Guglielmo, 2015). Because it is not easy to discern whether someone coming within 1.5 meters of someone else represents an intentional transgression, many behaviors that reflect COVID-19 non-adherence may lack the features of the type of intentional transgression to which human moral systems are attuned.

### Limitations on Study Design and Future Research

Our study design had several limitations that should be addressed by future research.

#### Single-Item Measures

Because the study survey assessed a diverse set of constructs (e.g., perceptions of others’ adherence, own adherence, anticipated shame for non-adherence, moral wrongness of non-adherence) for multiple scenarios, it was not feasible to use a lengthy measure for each construct for each scenario; doing so would have resulted in a long and unwieldy survey that induced participant fatigue and attrition. Previous research on several of the central constructs of interest has employed a similar design: a single-item measure of the construct for each scenario (e.g., Sznycer et al., 2018’s cross-cultural research on shame, DeScioli et al., 2011’s research on moral wrongness). Although some work (e.g., Loo, 2002) has expressed concerns about the psychometric validity of single-item scales, the items used in the current study exhibit high face validity, and multiple studies have empirically demonstrated that single-item scales tap the same construct as their lengthier counterparts (see Gardner et al., 1998; Yarkoni, 2010; Konstabel et al., 2017). Moreover, the ability of single items to tap the same construct as multi-item measures has not just been observed generally, but for shame specifically: the mean item-total correlations for multiple subscales of the Guilt and Shame Proneness scale (Cohen et al., 2011) exceed 0.70, indicating that any of several individual items would be psychometrically valid substitutes for longer scales (data available at https://osf.io/3wf4a/). In short, the single-item measures that we used followed precedents in the relevant literature, exhibited high face validity, and tapped constructs that specifically have been shown to be validly assessable via individual items. Nonetheless, further studies on the phenomena and relationships observed in the current study would benefit from employing longer measures with demonstrated psychometric validity, especially when researchers can afford the increased questionnaire length.

#### Generalizing Study Findings to a Global Scale

The current study’s sample was drawn exclusively from Australia. This may limit the cross-cultural generalizability of study findings. Australia’s relatively “loose” individualistic culture, for example, may offer a proximate explanation for the absence of a link between perceived social norms and anticipated shame for non-adherence. A pattern similar to one observed in the current study was recently observed in a set of studies based out of the Netherlands—a nation with a very “loose” culture (Gelfand et al., 2021). In these Netherlands-based studies, participants (*N* = 1142) did not increase conformity in response to high perceived pathogenic infection risk (van Leeuwen and Petersen, 2021). This contrasts with countries like Japan, which is a collectivist, tight culture in which people value norms of prosocial cooperation and are unlikely to engage in behavior that could result in ostracism (Böhm et al., 2016). In the context of the COVID-19 pandemic, nations with higher levels of cultural tightness had approximately 5 times fewer cases compared to countries with comparatively higher levels of cultural looseness (see Gelfand et al., 2021). This important proximate role of cultural tightness-looseness on COVID-19-related outcomes highlights the need for future work to investigate cross-cultural similarities and differences in the predictors of NPI adherence.

#### Individual Differences

Future work should also investigate the role of individual differences as predictors of adherence to NPIs. For example, slow life history strategists are characterized by a propensity to long-term planning and risk aversion (Del Giudice and Belsky, 2011). Consistent with this, individuals who pursue a slower life history strategy exhibit greater adherence to COVID-19 precautions (Corpuz et al., 2020). Theory and evidence also suggest that differential selection pressures shaped higher pathogen disgust and greater health-related concern in women relative to men (see Al-Shawaf and Lewis, 2013; Al-Shawaf et al., 2018; Luoto and Varella, 2021). Such differences may orient women to engage in a more cautious approach toward COVID-19 than men and be associated with sex differences in attitudes toward protective behaviors and adherence to NPIs (Dinić and Bodroža, 2021; Luoto and Varella, 2021). Given these important roles of individual differences in the context of the COVID-19 pandemic, future research should incorporate these and other individual differences into their investigations of adherence to NPIs (see Varella et al., 2021; see also Tybur et al., 2016). We also encourage the interested reader to consult evolutionary literature on why individuals in different phenotypic condition are expected to exhibit different responses to the same environmental inputs (e.g., Lewis, 2015; Lewis et al., 2018, 2020a,b; Lukaszewski et al., 2020).

#### Other NPIs

The analyses presented in the current study cannot address the issue of non-adherence to quarantine measures. As breaches of quarantine constitute a significant infection risk and have been frequently reported, future research is needed to understand the reasons why people do or do not follow quarantine protocols. One challenge that such research will face is that only a small proportion of the population is actually advised to quarantine. This presents a measurement challenge, as people who have not been advised to quarantine may report their current adherence as high (i.e., “I would quarantine if I had to”) or low (i.e., “I haven’t had COVID, so I haven’t quarantined”) depending on their interpretation of the question. To address this methodological challenge, future research could specifically target members of the population who are known to have had experience with quarantine.

#### Do Perceived Norms Actually Influence Adherence?

Because the present study was correlational, we cannot infer with certainty the causal direction, if any, of the observed relationship between perceived social norms and participants’ self-reported adherence. One possibility is that people simply associate with others who engage in similar behaviors, but are not actually influenced by others’ behavior. This alternative account would be plausible if participants considered just their own interpersonal milieu when answering questions about others’ adherence. However, the question we asked was: “What percentage of people *in your country* do you think follow the recommendations below?” [emphasis added]. The wording of the question was designed to prompt participants to consider a much larger reference group than just their immediate social circle. Nonetheless, there is research to suggest that individuals may exhibit an in-group bias in the context of pathogen risk wherein they disproportionately direct attention to and weight the actions of members of their in-group (see Navarrete and Fessler, 2006; see also Thornhill and Fincher, 2014). We therefore cannot conclusively rule out the possibility that participants used, as a reference group, just those individuals with whom they associate, despite being explicitly instructed to use country-wide levels of adherence as the point of reference.

Another possibility is that the statistical association between participants’ perceptions of others’ adherence and their own self-reported adherence resulted from participants engaging in socially desirable responding wherein they matched their own *reported* levels of adherence to their perceptions of others’ adherence. Those participants who perceived others as showing greater adherence may have had the greatest incentive to self-report similarly high levels of adherence, whereas participants who perceived others to not be adhering would have had less incentive to self-report high levels of adherence. Such socially desirable responding could lead to a statistical association between participants’ self-reported adherence and their perceptions of others’ adherence. However, this account does not appear to easily explain either (1) the relationship observed between perceived norms and perceptions of disease severity or (2) the indirect effect observed in the mediation model (and replicated in the alternative serial mediation model). This suggests that although socially desirable responding may have occurred in this study, it cannot account for the overall pattern of findings. Future studies using self-report measures of adherence to NPIs would nonetheless benefit from including a social desirability scale to control for socially desirable responding (Larson, 2019). More broadly, future research—especially experimental designs that manipulate perceived social norms—is needed to more conclusively establish the influence of social norms on adherence to public health measures during a pandemic.

### Implications of the Present Study

The findings from the present study, although preliminary, could have considerable implications in the ongoing fight against COVID-19 and future pandemics. The current findings suggest that public messaging campaigns designed to promote adherence to NPIs are more likely to be effective when they focus on “leading by example,” or what social psychologists have sometimes referred to as “social proof” (Cialdini, 2009). This also points toward the critical role that political leaders may play in fighting the pandemic—not just through their policy content and mandates (Luoto and Varella, 2021; Priesemann et al., 2021), but through their behaviors on display for the public eye. If, as the current study suggests, people use others’ behavior as an informative cue to the seriousness of the pandemic, then the behavior of political leaders, social influencers, and social movements—which are widely disseminated on public television and social media—may have a profound influence on people’s perception of the pandemic and, crucially, the actions they take in response to it (see also Haslam et al., 2021, for a discussion of the top-down influence of leader identity).

It is worth stressing the importance of this point. If the findings of the current study are robust, to act in the opposite way (i.e., modeling non-adherent behavior) could have the detrimental effect of promoting the flouting of NPI guidelines. If so, it is paramount that individuals with influence in the public sphere be mindful of the role of their own publicly observable behavior in combating pandemics.

## Data Availability Statement

The datasets presented in this study can be found in online repositories. The names of the repository/repositories and accession number(s) can be found below: https://osf.io/nsf2p/?view_only=b54137011201471aaebd857f47f57a19.

## Ethics Statement

The studies involving human participants were reviewed and approved by the Murdoch University Human Research Ethics Committee. The online survey explicitly asked participants for their consent to participate; participants were not allowed to access the study survey unless they clicked on a “Yes” button to indicate their consent to participate.

## Author Contributions

JN: conceptualization, writing—original draft, reviewing and editing, and visualization. KE: conceptualization, methodology, and data collection. AS: conceptualization and methodology. LA-S: conceptualization, methodology, and manuscript preparation. DL: conceptualization, supervision, writing—reviewing and editing. All authors contributed to the article and approved the submitted version.

## Conflict of Interest

The authors declare that the research was conducted in the absence of any commercial or financial relationships that could be construed as a potential conflict of interest.

## Footnotes

1 We implemented this using Model 4 in PROCESS with participant adherence as the criterion, perception of others’ adherence as the predictor, and anticipated shame, perceived moral wrongness of non-adherence, and perceived disease severity as mediators.

2 We encourage the interested reader to consult Krasnow (2017), who discusses the distinction between “inward-facing” and “outward-facing” moral mechanisms. In the serial mediation model, both inward-facing (i.e., designed to regulate one’s own behavior) and outward-facing (i.e., designed to regulate others’ behavior) facets of moral cognition may come into play. In this model, moral cognition regulates one’s own behavior—individuals may perceive non-adherence to risk harm to others, resulting in judgments of moral wrongness for non-adherence and thereby motivating adherence. However, this inward-facing function may itself be at least partly in the service of the outward-facing function of regulating others’ behavior: these judgments of moral wrongness may activate the shame program, whose principal function is to avoid and mitigate the costs of devaluation by others.

## References

[B1] Ackerman J. M. Hill S. E. Murray D. R. (2018). The behavioral immune system: current concerns and future directions. *Soc. Personal. Psychol. Compass* 12:e12371. 10.1111/spc3.12371

[B2] Al-Shawaf L. Lewis D. M. G. (2013). Exposed intestines and contaminated cooks: sex, stress, & satiation predict disgust sensitivity. *Pers. Individ. Diff.* 54 698–702. 10.1016/j.paid.2012.11.016

[B3] Al-Shawaf L. Lewis D. M. G. Buss D. M. (2018). Sex differences in disgust: why are women more easily disgusted than men? *Emot. Rev.* 10 149–160. 10.1177/1754073917709940

[B4] Al-Shawaf L. Lewis D. M. G. Wehbe Y. S. Buss D. M. (2019). “Context, environment, and learning in evolutionary psychology,” in *Encyclopedia of Evolutionary Psychological Science*, eds Shackelford T. K. Weekes-Shackelford V. A. (New York: Springer International Publishing), 1–12. 10.1007/978-3-319-16999-6_227-1

[B5] Anderson R. M. Heesterbeek H. Klinkenberg D. Hollingsworth T. D. (2020). How will country-based mitigation measures influence the course of the COVID-19 epidemic? *Lancet* 395 931–934. 10.1016/S0140-6736(20)30567-5 32164834 PMC7158572

[B6] Australian Bureau of Statistics. (2020). *Measuring the impacts of COVID-19, Mar-May 2020.* URL: https://www.abs.gov.au/articles/measuring-impacts-covid-19-mar-may-2020

[B7] Bavel J. J. V. Baicker K. Boggio P. S. Capraro V. Cichocka A. Cikara M. (2020). Using social and behavioural science to support COVID-19 pandemic response. *Nat. Hum. Behav.* 4 460–471. 10.1038/s41562-020-0884-z 32355299

[B8] Block R. Berg A. Lennon R. P. Miller E. L. Nunez-Smith M. (2020). African American adherence to COVID-19 public health recommendations. *Health Lit. Res. Pract.* 4 e166–e170. 10.3928/24748307-20200707-01 32926172 PMC7410494

[B9] Bocian K. Wojciszke B. (2014). Self-interest bias in moral judgments of others’ actions. *Personal. Soc. Psychol. Bull.* 40 898–909. 10.1177/0146167214529800 24743603

[B10] Böhm R. Betsch C. Korn L. (2016). Selfish-rational non-vaccination: experimental evidence from an interactive vaccination game. *J. Econ. Behav. Organ.* 131 183–195. 10.1016/j.jebo.2015.11.008

[B11] Cheng H. Y. Jian S. W. Liu D. P. Ng T. C. Huang W. T. Lin H. H. (2020). Contact tracing assessment of COVID-19 transmission dynamics in Taiwan and risk at different exposure periods before and after symptom onset. *JAMA Intern. Med.* 180 1156–1163. 10.1001/jamainternmed.2020.2020 32356867 PMC7195694

[B12] Christakis N. A. Fowler J. H. (2013). Social contagion theory: examining dynamic social networks and human behavior. *Stat. Med.* 32 556–577. 10.1002/sim.5408 22711416 PMC3830455

[B13] Cialdini R. (2009). *Influence. The Psychology of Persuasion* (Rev.ed). New York: HarperCollins.

[B14] Cohen T. Wolf S. Panter A. Insko C. (2011). Introducing the GASP scale: a new measure of guilt and shame proneness. *J. Personal. Soc. Psychol.* 100 947–966. 10.1037/a0022641 21517196

[B15] Corpuz R. D’Alessandro S. Adeyemo J. Jankowski N. Kandalaft K. (2020). Life history orientation predicts COVID-19 precautions and projected behaviors. *Front. Psychol* 11:1857. 10.3389/fpsyg.2020.01857 32793087 PMC7393224

[B16] Csibra G. Gergely G. (2007). Social learning and social cognition: the role of pedagogy. *Magyar Pszichologiai Szemle* 62 5–30.

[B17] Del Giudice M. Belsky J. (2011). “The development of life history strategies: toward a multi-stage theory,” in *The evolution of personality and individual differences*, eds Buss D. M. Hawley P. H. (Oxford: Oxford University Press), 154–176. 10.1093/acprof:oso/9780195372090.003.0006

[B18] DeScioli P. Kurzban R. (2013). A solution to the mysteries of morality. *Psychol. Bull.* 139 477–496. 10.1037/a0029065 22747563

[B19] DeScioli P. Christner J. Kurzban R. (2011). The omission strategy. *Psychol. Sci.* 22 442–446. 10.1177/0956797611400616 21372326

[B20] Dickie R. Rasmussen S. Cain R. Williams L. Mackay W. (2018). The effects of perceived social norms on handwashing behaviour in students. *Psychol. Health Med.* 23 154–159. 10.1080/13548506.2017.1338736 28592138

[B21] Dinić B. M. Bodroža B. (2021). COVID-19 protective behaviors are forms of prosocial and unselfish behaviors. *Front. Psychol.* 12:647710. 10.3389/fpsyg.2021.647710 33897553 PMC8062771

[B22] Fritz M. S. Mackinnon D. P. (2007). Required sample size to detect the mediated effect. *Psychol. Sci.* 18 233–239. 10.1111/j.1467-9280.2007.01882.x 17444920 PMC2843527

[B23] Gardner D. G. Cummings L. L. Dunham R. B. Pierce J. L. (1998). Single-item versus multiple-item measurement scales: an empirical comparison. *Educ. Psychol. Meas.* 58 898–915. 10.1177/0013164498058006003

[B24] Gelfand M. J. Jackson J. C. Pan X. Nau D. Pieper D. Denison E. (2021). The relationship between cultural tightness-looseness and COVID-19 cases and deaths: a global analysis. *Lancet Planet. Health* 5 e135–e144. 10.1016/S2542-5196(20)30301-6 33524310 PMC7946418

[B25] Gilles I. Bangerter A. Clémence A. Green E. G. T. Krings F. Staerklé C. (2011). Trust in medical organizations predicts pandemic (H1N1) 2009 vaccination behavior and perceived efficacy of protection measures in the Swiss public. *Eur. J. Epidemiol.* 26 203–210. 10.1007/s10654-011-9577-2 21476079

[B26] Greene J. D. (2001). An fMRI investigation of emotional engagement in moral judgment. *Science* 293 2105–2108. 10.1126/science.1062872 11557895

[B27] Guglielmo S. (2015). Moral judgment as information processing: an integrative review. *Front. Psychol.* 6:1637. 10.3389/fpsyg.2015.01637 26579022 PMC4626624

[B28] Habib H. (2020). Has Sweden’s controversial covid-19 strategy been successful? *BMJ* 369:m2376. 10.1136/bmj.m2376 32532807

[B29] Haslam S. A. Steffens N. K. Reicher S. D. Bentley S. V. (2021). Identity Leadership in a Crisis: a 5R Framework for Learning from Responses to COVID-19. *Soc. Issues Policy Rev.* 15 35–83. 10.1111/sipr.12075 33821168 PMC8013601

[B30] Hayes A. (2017). *Introduction To Mediation, Moderation, And Conditional Process Analysis: A Regression Based Approach* (2^*nd*^ ed.). New York: Guilford Press.

[B31] Hayes A. F. (2009). Beyond Baron and Kenny: statistical mediation analysis in the new millennium. *Commun. Monogr.* 76 408–420. 10.1080/03637750903310360

[B32] Huff H. V. Singh A. (2020). Asymptomatic transmission during the coronavirus disease 2019 pandemic and implications for public health strategies. *Clin. Infect. Dis.* 71 2752–2756. 10.1093/cid/ciaa654 32463076 PMC7314132

[B33] Kang H. (2013). The prevention and handling of the missing data. *Korean J. Anesthesiol.* 64 402–406. 10.4097/kjae.2013.64.5.402 23741561 PMC3668100

[B34] Konstabel K. Lönnqvist J.-E. Leikas S. Velázquez R. G. Qin H. Verkasalo M. (2017). Measuring single constructs by single items: constructing an even shorter version of the “Short Five” personality inventory. *PLoS One* 12:e0182714. 10.1371/journal.pone.0182714 28800630 PMC5553894

[B35] Krasnow M. M. (2017). An evolutionarily informed study of moral psychology. In *Moral Psychology: A Multidisciplinary Guide*, eds Voyer B. Tarantola T. (USA: Springer).

[B36] Larson R. B. (2019). Controlling social desirability bias. *Int. J. Mark. Res.* 61 534–547. 10.1177/1470785318805305

[B37] Lennon R. P. Sakya S. M. Miller E. L. Snyder B. Yaman T. Zgierska A. E. (2020). Public intent to comply with COVID-19 public health recommendations. *Health Lit. Res. Pract.* 4 e161–e165. 10.3928/24748307-20200708-01 32926171 PMC7410495

[B38] Lewis D. M. G. (2015). Evolved individual differences: advancing a condition-dependent model of personality. *Pers. Individ. Diff.* 84 63–72. 10.1016/j.paid.2014.10.013

[B39] Lewis D. M. G. Al-Shawaf L. Buss D. M. (2020a). “Evolutionary personality psychology,” in *The Cambridge Handbook of Personality Psychology*, 2nd Edn, eds Corr P. Matthews G. (Cambridge: Cambridge University Press), 223–234. 10.1017/9781108264822.022

[B40] Lewis D. M. G. Al-Shawaf L. Conroy-Beam D. Asao K. Buss D. M. (2017). Evolutionary psychology: a how-to guide. *Am. Psychol*. 72 353–373. 10.1037/a0040409 28481582

[B41] Lewis D. M. G. Al-Shawaf L. Janiak M. Akunebu S. (2018). Integrating molecular genetics and evolutionary psychology: sexual jealousy and the androgen receptor (AR) gene. *Pers. Individ. Diff.* 120 276–282. 10.1016/j.paid.2016.11.021

[B42] Lewis D. M. G. Al-Shawaf L. Thompson M. B. Buss D. M. (2020b). “Evolved psychological mechanisms,” in *SAGE Handbook of Evolutionary Psychology*, ed. Shackelford T. K. (California: Sage), 96–119. 10.4135/9781529739442.n6

[B43] Loo R. (2002). A caveat on using single-item versus multiple-item scales. *J. Manag. Psychol.* 17 68–75. 10.1108/02683940210415933

[B44] Lu G. Razum O. Jahn A. Zhang Y. Sutton B. Sridhar D. (2021). COVID-19 in Germany and China: mitigation versus elimination strategy. *Glob. Health Action* 14:1875601. 10.1080/16549716.2021.1875601 33472568 PMC7833051

[B45] Lukaszewski A. W. Lewis D. M. G. Durkee P. K. Sell A. N. Sznycer D. Buss D. M. (2020). An adaptationist framework for personality science. *Eur. J. Personal.* 34 1151–1174. 10.1002/per.2292

[B46] Luoto S. Varella A. C. (2021). Pandemic leadership: sex differences and their evolutionary-developmental origins. *Front. Psychol.* 12:633862. 10.3389/fpsyg.2021.633862 33815218 PMC8015803

[B47] Markowitz E. M. Shariff A. F. (2012). Climate change and moral judgement. *Nat. Clim. Chang.* 2 243–247. 10.1038/nclimate1378

[B48] Miller D. T. Prentice D. A. (1996). “The construction of social norms and standards,” in *Social Psychology: Handbook Of Basic Principles*, eds Higgins E. T. Kruglanski A. W. (New York: Guilford Press), 799–829.

[B49] Mitchell T. Dee D. L. Phares C. R. Lipman H. B. Gould L. H. Kutty P. (2011). Non-pharmaceutical interventions during an outbreak of 2009 pandemic influenza a (H1N1) virus infection at a large public university, April-May 2009. *Clin. Infect. Dis.* 52 S138–S145. 10.1093/cid/ciq056 21342886

[B50] Navarrete C. D. Fessler D. M. T. (2006). Disease avoidance and ethnocentrism: the effects of disease vulnerability and disgust sensitivity on intergroup attitudes. *Evol. Hum. Behav.* 27 270–282. 10.1016/j.evolhumbehav.2005.12.001

[B51] Nezlek J. B. Wesselmann E. D. Wheeler L. Williams K. D. (2012). Ostracism in everyday life. *Group Dyn.* 16 91–104. 10.1037/a0028029 26267126

[B52] Ölcer S. Yilmaz-Aslan Y. Brzoska P. (2020). Lay perspectives on social distancing and other official recommendations and regulations in the time of COVID-19: a qualitative study of social media posts. *BMC Public Health* 20:963. 10.1186/s12889-020-09079-5 32560716 PMC7303937

[B53] Perreau De Pinninck A. Sierra C. Schorlemmer M. (2007). “Distributed norm enforcement via ostracism,” in *Coordination, Organizations, Institutions, and Norms in Agent Systems III*, eds Sichman J. S. Padget J. Ossowski S. Noriega P. (Berlin: Springer), 301–315. 10.1007/978-3-540-79003-7_22

[B54] Petersen E. Koopmans M. Go U. Hamer D. H. Petrosillo N. Castelli F. (2020). Comparing SARS-CoV-2 with SARS-CoV and influenza pandemics. *Lancet Infect. Dis.* 20 e238–e244. 10.1016/S1473-3099(20)30484-9 32628905 PMC7333991

[B55] Pfattheicher S. Strauch C. Diefenbacher S. Schnuerch R. (2018). A field study on watching eyes and hand hygiene compliance in a public restroom. *J. Appl. Soc. Psychol.* 48 188–194. 10.1111/jasp.12501

[B56] Priesemann V. Brinkmann M. M. Ciesek S. Cuschieri S. Czypionka T. Giordano G. (2021). Calling for pan-European commitment for rapid and sustained reduction in SARS-CoV-2 infections. *Lancet* 397 92–93. 10.1016/S0140-6736(20)32625-8 33347811 PMC7833270

[B57] Raza A. Raza I. Drake T. M. Sadar A. B. Adil M. Baluch F. (2017). The efficiency, accuracy and acceptability of smartphone-delivered data collection in a low-resource setting – A prospective study. *Int. J. Surg.* 44 252–254. 10.1016/j.ijsu.2017.06.081 28676384

[B58] Robertson T. E. Delton A. W. Klein S. B. Cosmides L. Tooby J. (2014). Keeping the benefits of group cooperation: domain-specific responses to distinct causes of social exclusion. *Evol. Hum. Behav.* 35 472–480. 10.1016/j.evolhumbehav.2014.06.006

[B59] Robertson T. E. Sznycer D. Delton A. W. Tooby J. Cosmides L. (2018). The true trigger of shame: social devaluation is sufficient, wrongdoing is unnecessary. *Evol. Hum. Behav.* 39 566–573. 10.1016/j.evolhumbehav.2018.05.010

[B60] Rudert S. C. Ruf S. Greifeneder R. (2019). Whom to punish? How observers sanction norm-violating behavior in ostracism situations. *Eur. J. Soc. Psychol.* 50 376–391. 10.1002/ejsp.2606

[B61] Scott-Phillips T. C. Dickins T. E. West S. A. (2011). Evolutionary theory and the ultimate–proximate distinction in the human behavioral sciences. *Perspect. Psychol. Sci.* 6 38–47. 10.1177/1745691610393528 26162114

[B62] Seitz B. M. Aktipis A. Buss D. M. Alcock J. Bloom P. Gelfand M. (2020). The pandemic exposes human nature: 10 evolutionary insights. *Proc. Natl. Acad. Sci. U. S. A.* 117 27767–27776. 10.1073/pnas.2009787117 33093198 PMC7668083

[B63] Shultz J. M. Baingana F. Neria Y. (2015). The 2014 ebola outbreak and mental health. *J. Am. Med. Assoc.* 313:567. 10.1001/jama.2014.17934 25532102

[B64] Spoor J. Williams K. (2007). “The evolution of an ostracism detection system,” in *Evolution and the Social Mind*, eds Forgas J. P. Haselton M. G. von Hippel W. (United Kingdom: Psychology Press), 279–292.

[B65] Steelfisher G. K. Blendon R. J. Ward J. R. Rapoport R. Kahn E. B. Kohl K. S. (2012). Public response to the 2009 influenza A H1N1 pandemic: a polling study in five countries. *Lancet Infect. Dis.* 12 845–850. 10.1016/s1473-3099(12)70206-2 23041199

[B66] Sznycer D. (2019). Forms and functions of the self-conscious emotions. *Trends Cogn. Sci.* 23 143–157. 10.1016/j.tics.2018.11.007 30583948

[B67] Sznycer D. Tooby J. Cosmides L. Porat R. Shalvi S. Halperin E. (2016). Shame closely tracks the threat of devaluation by others, even across cultures. *Proc. Natl. Acad. Sci. U. S. A.* 113 2625–2630. 10.1073/pnas.1514699113 26903649 PMC4790975

[B68] Sznycer D. Xygalatas D. Agey E. Alami S. An X.-F. Ananyeva K. I. (2018). Cross-cultural invariances in the architecture of shame. *Proc. Natl. Acad. Sci. U. S. A.* 115 9702–9707. 10.1073/pnas.1805016115 30201711 PMC6166838

[B69] Taylor S. (2019). *The Psychology Of Pandemics: Preparing For The Next Global Outbreak Of Infectious Disease.* Cambridge: Cambridge Scholars Publishing.

[B70] Thornhill R. Fincher C. L. (2014). The parasite-stress theory of sociality, the behavioral immune system, and human social and cognitive uniqueness. *Evol. Behav. Sci.* 8 257–264. 10.1037/ebs0000020

[B71] Tindale L. C. Stockdale J. E. Coombe M. Garlock E. S. Lau W. Y. V. Saraswat M. (2020). Evidence for transmission of COVID-19 prior to symptom onset. *Elife* 9:e57149. 10.7554/eLife.57149 32568070 PMC7386904

[B72] Tybur J. M. Inbar Y. Aarøe L. Barclay P. Barlow F. K. De Barra M. (2016). Parasite stress and pathogen avoidance relate to distinct dimensions of political ideology across 30 nations. *Proc. Natl. Acad. Sci. U. S. A.* 113 12408–12413. 10.1073/pnas.1607398113 27791090 PMC5098626

[B73] van Leeuwen F. Petersen M. (2021). Pathogen avoidance and conformity: salient infectious disease does not increase conformity. *PsyArXiv* [preprint].

[B74] Varella M. A. C. Luoto S. Soares R. B. S. Valentova J. V. (2021). COVID-19 pandemic on fire: evolved propensities for nocturnal activities as a liability against epidemiological control. *Front. Psychol.* 12:646711. 10.3389/fpsyg.2021.646711 33828510 PMC8019933

[B75] Vygotsky L. S. (1978). *Mind In Society: The Development Of Higher Psychological Processes.* Cambridge: Harvard University Press.

[B76] Wang W. Wu Q. Yang J. Dong K. Chen X. Bai X. (2020). Global, regional, and national estimates of target population sizes for COVID-19 vaccination: descriptive study. *BMJ* 371:m4704. 10.1136/bmj.m4704 PMC7736995 33323388

[B77] Wesselmann E. D. Nairne J. S. Williams K. D. (2012). An evolutionary social psychological approach to studying the effects of ostracism. *J. Soc. Evol. Cult. Psychol.* 6 309–328. 10.1037/h0099249

[B78] Williams K. D. (2007). Ostracism: the kiss of social death. *Soc. Personal. Psychol. Compass* 1 236–247. 10.1111/j.1751-9004.2007.00004.x

[B79] Williams K. D. Shore W. J. Grahe J. E. (1998). The silent treatment: perceptions of its behaviors and associated feelings. *Group Process. Intergroup Relat.* 1 117–141. 10.1177/1368430298012002

[B80] Wilson S. (2020). Pandemic leadership: lessons from New Zealand’s approach to COVID-19. *Leadership* 16 279–293. 10.1177/1742715020929151

[B81] World Health Organization [WHO]. (2020a). *WHO Director-General’s Opening Remarks at the Media Briefing on COVID-19 - 11 March 2020.* URL: https://www.who.int/dg/speeches/detail/who-director-general-s-opening-remarks-at-the-media-briefing-on-covid-19—11-march-2020

[B82] World Health Organization [WHO]. (2020b). *WHO Coronavirus Disease (COVID-19) dashboard.* URL: https://covid19.who.int/

[B83] World Health Organization [WHO]. (2020c). *Q&A on Coronavirus (COVID-19).* URL: https://www.who.int/emergencies/diseases/novel-coronavirus-2019/question-and-answers-hub/q-a-detail/q-a-coronaviruses

[B84] World Health Organization [WHO]. (2020d). *Coronavirus Disease (Covid-19) Advice For The Public.* URL: https://www.who.int/emergencies/diseases/novel-coronavirus-2019/advice-for-public#:∼:text=If COVID-19 is spreading, a bent elbow or tissue

[B85] World Health Organization Writing Group. (2012). Nonpharmaceutical interventions for pandemic influenza, national and community measures. *Emerg. Infect. Dis.* 12 88–94. 10.3201/eid1201.051371 16494723 PMC3291415

[B86] Xu S. Li Y. (2020). Beware of the second wave of COVID-19. *Lancet* 395 1321–1322. 10.1016/S0140-6736(20)30845-X 32277876 PMC7194658

[B87] Yarkoni T. (2010). The abbreviation of ersonality, or how to measure 200 Personality Scales with 200 items. *J. Res. Personal.* 44 180–198. 10.1016/j.jrp.2010.01.002 20419061 PMC2858332

[B88] Yong J. C. Choy B. K. C. (2021). Noncompliance with safety guidelines as a free-riding strategy: an evolutionary game-theoretic approach to cooperation during the COVID-19 pandemic. *Front. Psychol.* 12:646892. 10.3389/fpsyg.2021.646892 33796057 PMC8008110

